# Effect of reconstruction parameters on cone beam CT trabecular bone microstructure quantification in sheep

**DOI:** 10.1186/s12903-020-1035-7

**Published:** 2020-02-10

**Authors:** Aso Muhammad Ali Muhammad, Norliza Ibrahim, Rohana Ahmad, Muhammad Khan Asif, Zamri Radzi, Zuraiza Mohamad Zaini, Hairil Rashmizal Abdul Razak

**Affiliations:** 1 Department of Oral and Maxillofacial Clinical Sciences, Faculty of Dentistry, University of Malaya, 50603 Kuala Lumpur, Malaysia; 2 Faculty of Dentistry, Universiti Teknologi MARA, Sungai Buloh Campus, Jalan Hospital 47000 Sungai Buloh, Shah Alam, Selangor Malaysia; 3 Department of Paediatric Dentistry and Orthodontics, Faculty of Dentistry, University of Malaya, 50603 Kuala Lumpur, Malaysia; 4 Centre for Diagnostic Nuclear Imaging, Universiti Putra Malaysia, 43400 Serdang, Selangor Malaysia

**Keywords:** CBCT, Trabecular bone, Reconstruction parameters, Threshold value

## Abstract

**Background:**

Cone Beam Computed Tomography (CBCT) is a reliable radiographic modality to assess trabecular bone microarchitecture. The aim of this study was to determine the effect of CBCT image reconstruction parameters, namely, the threshold value and reconstruction voxel size, on trabecular bone microstructure assessment.

**Methods:**

Five sectioned maxilla of adult Dorper male sheep were scanned using a CBCT system with a resolution of 76 μm^3^ (Kodak 9000). The CBCT images were reconstructed using different reconstruction parameters and analysed. The effect of reconstruction voxel size (76, 100 and 200 μm^3^) and threshold values (±15% from the global threshold value) on trabecular bone microstructure measurement was assessed using image analysis software (CT analyser version 1.15).

**Results:**

There was no significant difference in trabecular bone microstructure measurement between the reconstruction voxel sizes, but a significant difference (Tb.*N* = 0.03, Tb.Sp = 0.04, Tb.Th = 0.01, BV/TV = 0.00) was apparent when the global threshold value was decreased by 15%.

**Conclusions:**

Trabecular bone microstructure measurements are not compromised by changing the CBCT reconstruction voxel size. However, measurements can be affected when applying a threshold value of less than 15% of the recommended global value.

## Background

Assessment of trabecular bone microstructure from CBCT images prior to implant surgery is a growing treatment planning protocol to ensure successful placement and clinical longevity of the dental implants [1]. This is because the trabecular bone microstructure has been shown to have major effects on primary stability during implant placement [2, 3]. In addition, primary stability can be accurately predicted based on the status of the trabecular bone microarchitecture prior to implant placement [4]. Nevertheless, the assessment of trabecular parameters from CBCT images may be compromised by the reconstruction parameters that are used for image segmentation, such as the reconstruction voxel size and threshold value.

Reconstruction voxel size is the voxel size that is used to reconstruct an image of raw data. A larger image reconstruction voxel size than the actual image acquisition voxel size is usually used to reduce the reconstruction time and to reduce computational expenses [5]. This is used in some practices such as analysing large-scale finite element models using a micro- CT-based image [5–7]. However, it is presumed that the image accuracy will be decreased when using a larger reconstruction voxel size than the acquisition voxel size [8]. The effect of CBCT reconstruction voxel size has focused on image quality [9] and 3D measurement of dental morphology [10]. However, the effect on trabecular bone microstructure measurements remains unknown.

Segmentation is a process of separating images to either bone or other soft tissue structures which can be affected by the threshold values [11]. Many studies have used μCT to investigate the effect of threshold values on trabecular microstructure parameters such as trabecular number (Tb.N), thickness (Tb.Th), spacing (Tb.Sp) and bone volume fraction (BV/TV) of small animal models [12]. Although studies on scanning parameters are abundant in large animal models, the effect of reconstruction parameters using CBCT are scarce. Hence, as it has been suggested that CBCT can be used for trabecular bone measurement to aid surgical treatment planning, the CBCT reconstruction parameters that affect these measurements should be investigated.

Threshold values are commonly used for segmentation as it is the most straightforward and feasible segmentation method specifically when measuring bone volume [13]. Therefore, its effect on trabecular quantification is also investigated in this study. The findings of this study will provide clinicians with an evidence-based guideline in deciding the suitable reconstruction voxel size and threshold values to be used for their CBCT image reconstruction prior to trabecular bone microstructure analysis.

## Methods

Five sectioned maxillae of adult Dorper male sheep (age ranging from 20 to 48 months and body weight 50–60 kg) were obtained from the Animal Experimental Unit, Faculty of Veterinary Medicine, University Putra Malaysia (UPM), Malaysia following approval from the Institutional Animal Care and Use Committee (IACUC), UPM (No. UPM/IACUC/AUP-R031). The samples were imaged using a CBCT system (Carestream 9000, Kodak) at the Division of Oral Radiology, Faculty of Dentistry, University of Malaya, Kuala Lumpur, Malaysia. The selected scanning parameters for CBCT were 76 μm^3^ for the voxel size, 4 × 4 cm FOV and 360° arm rotation. Images were acquired at 65kv, 6 mA and 10.8 s. The samples were fitted in a cylinder-shaped plastic container to reduce any possible movement during scanning. The images were subsequently converted into BMP and imported into DataViewer (v 1.5, SkyScan, Kontich, Belgium) to obtain the sagittal view for image processing and analyses in CTAnalyser software (v 1.15, SkyScan, Kontich, Belgium).

### The effect of CBCT reconstruction voxel size on trabecular bone microstructure assessment

**Fig. 1:**
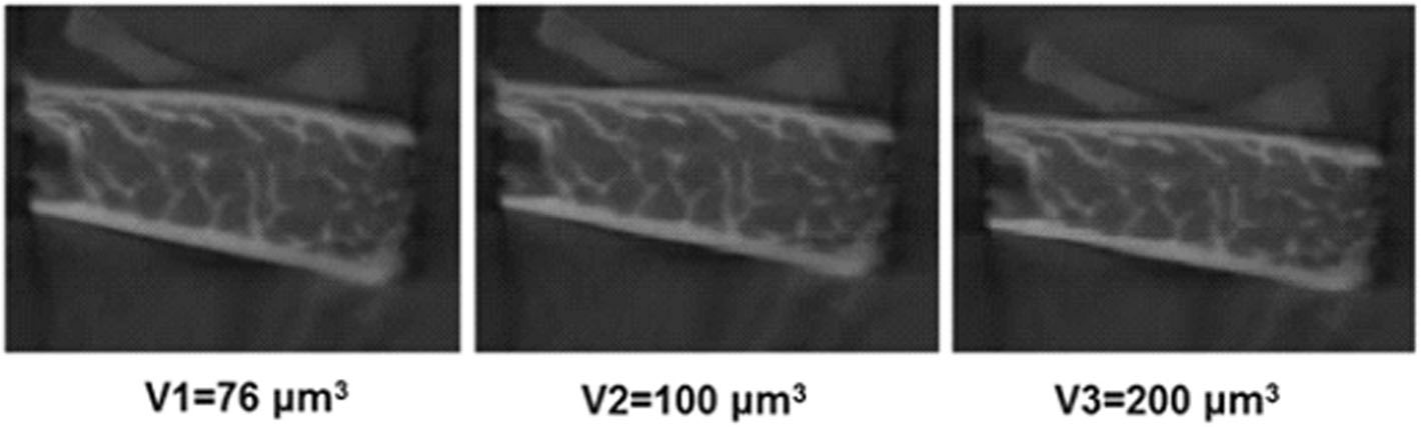
Three reconstructed images using voxel size of 76 μm^3^ (V1), 100 μm^3^ (V2) and 200 μm^3^ (V3) were binarised and analysed using CT-Analyser software

The images were reconstructed using three different voxel sizes (76, 100, and 200 μm^3^) resulting in three different datasets. For the first dataset, a ROI was selected (14 × 8 mm) and reconstructed using a voxel size of 76 μm^3^. Then, the same ROI was used to reconstruct the other 2 datasets using a voxel size of 100 and 200 μm^3^, respectively (Fig. 1). Thus, three sets of ROI were obtained which were thresholded and binarized using a global threshold value (threshold value 82). The global threshold value was automatically generated by CTAnalyser software (v 1.15, SkyScan, Kontich, Belgium). This global threshold value was used as the optimal threshold value in this study. Later, trabecular bone analysis was performed using CTAnalyser software (v 1.15, SkyScan, Kontich, Belgium) to examine the effect of various reconstruction voxel sizes on the assessment of trabecular bone parameters (Tb.N, Tb.Th, Tb.Sp and BV/TV).

### The effect of CBCT threshold value on trabecular bone microstructure assessment

**Fig. 2:**
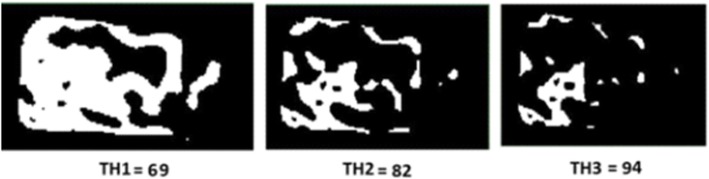
Images were thresholded + 15% from the global threshold value (69, 82: global threshold value and 94) and binarised in the CTAnalyser software

The effect of different range of threshold values on the CBCT datasets was assessed using the first dataset as a reference. Three datasets were created by varying the global threshold value as follows: (Dataset A) decreasing the global selected threshold value by 15%, (Dataset B) and (Dataset C) increasing the global threshold value by 15% (Fig. 2). The images from all 3 datasets were then exported into CTAnalyser software (v 1.15, SkyScan, Kontich, Belgium) for the selection of the ROI. Three-dimensional analyses were then performed on all three datasets to assess the trabecular bone parameters (Tb.N, Tb.Th, Tb.Sp and BV/TV).

## Statistical analyses

The assessments were performed twice by two examiners with a minimum interval of one week between the two measurements. Intraclass correlation coefficient (ICC) was employed to examine the intra-observer reliability and inter-rater agreement in reproducing the measurements. The trabecular bone microstructure parameters that were assessed include trabecular number (Tb.N), thickness (Tb.Th), separation (Tb.Sp) and bone volume fraction (BV/TV). One-way ANOVA was used to assess the differences obtained from three reconstruction voxel sizes and threshold values. Additionally, the Bonferroni test was used as a post hoc test to examine the significant differences between threshold values.

## Results

### The effect of reconstruction voxel size on the assessment of trabecular bone microstructure

The intra-observer reproducibility (> 0.91) and inter-rater agreement (> 0.80) of trabecular bone microstructure measurements were excellent for all parameters (Tables 1 and 2). One-way ANOVA showed no significant difference between the three reconstruction voxel sizes in all trabecular bone microstructures (Tb.N, *p* = 0.579; Tb.Th, *p* = 0.095; Tb.Sp, *p* = 0.131; BV/TV, *p* = 0.908), as shown in Table 3.

**Table 1:** Intraobserver reliability for different reconstruction voxel sizes using the intraclass correlation coefficient (ICC)

	V1 = 76 μm^3^	V2 = 100 μm^3^	V3 = 200 μm^3^
Tb.N	0.991	0.986	0.977
Tb.Sp	0.912	0.973	0.989
Tb.Th	0.981	0.989	0.996
BV/TV	0.994	0.999	0.999

Trabecular number (Tb.N), separation (Tb.Sp), thickness (Tb.Th), and bone volume fraction (BV/TV)

V = Voxel size

*p < 0.05

**Table 2:** Inter-rater agreement for different reconstruction voxel sizes using the intraclass correlation coefficient (ICC)

	V1 = 76 μm^3^	V2 = 100 μm^3^	V3 = 200 μm^3^
Tb.N	0.823	0.840	0.813
Tb.Sp	0.804	0.853	0.876
Tb.Th	0.883	0.819	0.837
BV/TV	0.829	0.871	0.845

Trabecular number (Tb.N), separation (Tb.Sp), thickness (Tb.Th), and bone volume fraction (BV/TV)

V = Voxel size

*p < 0.05

**Table 3:** The mean and standard deviation (SD) of trabecular microstructure measurements analyzed using One Way ANOVA for different reconstruction voxel size

Voxel size	n	Trabecular number Tb.N (μm^− 1^)			Trabecular spacing Tb.Sp (μm)			Trabecular thickness Tb.Th (μm)			Bone Volume/Tissue volume BV/TV %		
		Mean	SD	*p*	Mean	SD	*p*	Mean	SD	*p*	Mean	SD	*p*
76 μm^3^	5	0.0153	0.007	0.579	17.6388	2.040	0.095	6.8204	1.832	0.131	11.4783	8.212	0.908
100 μm^3^	5	0.0168	0.008		16.8204	2.206		6.1350	1.966		11.6022	8.817	
200 μm^3^	5	0.0204	0.007		14.4790	2.230		4.3543	1.676		9.6478	6.309	

*p < 0.05

### The effect of different threshold values on trabecular bone microstructure measurements

The reproducibility of trabecular bone microstructure measurements revealed excellent intra-observer reliability (> 0.91) and inter-rater agreement (> 0.82) when using different threshold values (Tables 4 and 5). One-way ANOVA showed significant differences between the three tested threshold values. Bonferroni Post-Hoc analyses with pair-wise multiple comparisons were performed to test the difference between the three different threshold values. A significant difference was observed (Tb.*N* = 0.03, Tb.Sp = 0.04, Tb.Th = 0.01, BV/TV = 0.00) when the threshold value was decreased by 15% from the global value. However, the increase in the threshold value (Th: 90) from the global value (Th: 82) had no significant (*p* >0.05) effect on the trabecular bone measurements (Table 6).

**Table 4:** Intraobserver reliability for different threshold values using intraclass correlation coefficient (ICC)

	Th 1 = 73	Th 2 = 82	Th 3 = 90
Tb.N	0.988	0.991	0.994
Tb.Sp	0.996	0.912	0.975
Tb.Th	0.998	0.981	0.973
BV/TV	0.971	0.994	0.985

Trabecular number (Tb.N), separation (Tb.Sp), thickness (Tb.Th), bone volume fraction (BV/TV) and threshold value (Th)

**Table 5:** Inter-rater agreement for different threshold values using intraclass correlation coefficient (ICC)

	Th 1 = 73	Th 2 = 82	Th 3 = 90
Tb.N	0.870	0.853	0.824
Tb.Sp	0.857	0.841	0.872
Tb.Th	0.865	0.821	0.854
BV/TV	0.837	0.860	0.883

Trabecular number (Tb.N), separation (Tb.Sp), thickness (Tb.Th), bone volume fraction (BV/TV) and threshold value (Th)

**Table 6:** The mean and standard deviation (SD) of trabecular microstructural measurements analyzed using One Way ANOVA for different threshold values

Threshold value	n	Trabecular number Tb.N (μm^−1^)			Trabecular spacing Tb.Sp (μm)			Trabecular thickness Tb.Th (μm)			Bone Volume/Tissue volume BV/TV %		
		Mean	SD	*P*	Mean	SD	*p*	Mean	SD	*p*	Mean	SD	*p*
73	5	0.0264	0.00385		14.4607	1.49057		12.2463	3.12846		32.7632	10.87921	
82				0.033			0.037			0.007			0.004
90				0.000			0.001			0.000			0.000
82	5	0.0153	0.00784		17.6388	2.04041		6.8204	1.83221		11.4783	8.21205	
73				0.033			0.037			0.007			0.004
90				0.082			0.190			0.381			0.404
90	5	0.0059	0.00518		19.8544	1.55221		4.4882	1.42399		3.2503	3.42096	
73				0.000			0.001			0.000			0.000
82				0.82			0.190			0.381			0.404

*p < 0.05

## Discussion

The enhanced resolution of cone-beam computed tomography (CBCT) images has significantly improved the measurement of trabecular bone microstructure [14, 15]. Unlike scanning parameters (voxel size, field of view and scanning rotation) [16], the effect of CBCT reconstruction parameters (reconstruction voxel size and threshold value) on diagnostic accuracy of trabecular bone microstructure has not been reported [17, 18].

The accuracy of micro-CT images in measuring trabecular bone microstructure can be compromised by the reconstruction voxel size [8, 19, 20]. Previous micro-CT studies have reported that the image quality cannot be improved by using a smaller reconstruction voxel size after originally scanning the sample using a large voxel size [8, 21]. This is due to an increase in image noise (9). Therefore, in the current study, CBCT images that were scanned using 76 μm^3^ were reconstructed using larger voxel sizes (100 and 200 μm^3^). The findings indicated no differences in trabecular bone measurements between different reconstructed images. However, our results differ to those reported in previously studies [8, 21] as the scanning voxel size (76 μm^3^) is almost 4 times larger than was used in micro-CT studies (21 μm^3^). It was demonstrated in micro-CT studies that only certain trabecular bone parameters namely BV/TV and Tb.Th are compromised by scanning parameters when reconstructing the images of 21 μm^3^ using larger voxel sizes (50 and 110 μm^3^) [8]. This is due to the fact that the trabecular bone parameters are significantly affected by the scanning voxel size rather than the reconstruction voxel size. However, this effect can only be observed when the difference between the scan and reconstruction voxel is very large [21]. The results of the present study showed that trabecular bone microstructure measurements are not influenced by CBCT reconstruction voxel size, although it is important to note other CBCT systems with different scanning and reconstruction parameters may generate different results.

Threshold values may influence the analysis of trabecular microstructure parameters in micro-CT images [22–24]. Similarly, our study of CBCT images showed that a reducing the threshold value by 15% had a significant effect on all trabecular bone microstructure parameters (Tb.*N* = 0.03, Tb.Sp = 0.04, Tb.Th = 0.01, BV/TV = 0.00). However, increasing the threshold value from the global value had no significant (*p* < 0.05) effect on trabecular bone measurements (Table 4). The deviation of trabecular bone measurements might be due to partial volume effects that might alter the layers of voxels from the trabecular surface [25]. However, Tb.N measurements are insensitive to threshold variation if the selected threshold values are within the range of realistic values.

However, some limitations should be noted. In this study, the samples were not assessed using micro CT images due to computational constrains. Although strong correlations between micro CT and CBCT trabecular bone measurements have been largely described [26–28], the comparison in varying the reconstruction parameters is highly recommended to further validate the accuracy of the current findings.

## Conclusion

This study showed that trabecular bone microstructure measurements are not compromised by changing the CBCT reconstruction voxel size. However, measurements can be affected when applying a threshold value less than 15% of the recommended global value.

## Data Availability

The datasets used and/or analysed during the current study are available from the corresponding author on reasonable request.

## Abbreviations

BV/TV: Bone volume fraction
CBCT: Cone beam computed tomography
Micro-CT: Micro- computed tomography
Tb.N: Trabecular number
Tb.Sp: Trabecular spacing
Tb.Th: Trabecular thickness
